# CPI motif interaction is necessary for capping protein function in cells

**DOI:** 10.1038/ncomms9415

**Published:** 2015-09-28

**Authors:** Marc Edwards, Patrick McConnell, Dorothy A. Schafer, John A. Cooper

**Affiliations:** 1 Department of Cell Biology and Physiology, Washington University School of Medicine, St Louis, Missouri 63110-1093, USA; 2 Departments of Biology and Cell Biology, University of Virginia, Charlottesville, Virginia 22904-4328, USA

## Abstract

Capping protein (CP) has critical roles in actin assembly *in vivo* and *in vitro*. CP binds with high affinity to the barbed end of actin filaments, blocking the addition and loss of actin subunits. Heretofore, models for actin assembly in cells generally assumed that CP is constitutively active, diffusing freely to find and cap barbed ends. However, CP can be regulated by binding of the ‘capping protein interaction' (CPI) motif, found in a diverse and otherwise unrelated set of proteins that decreases, but does not abolish, the actin-capping activity of CP and promotes uncapping in biochemical experiments. Here, we report that CP localization and the ability of CP to function in cells requires interaction with a CPI-motif-containing protein. Our discovery shows that cells target and/or modulate the capping activity of CP via CPI motif interactions in order for CP to localize and function in cells.

## Abstract (web summary)

Capping protein regulates actin filament dynamics by binding to barbed ends and preventing their growth. Edwards *et al*. show that capping protein also requires interactions with proteins containing a capping protein interaction motif to promote its proper localization and regulation of actin dynamics.

## Graphical abstract

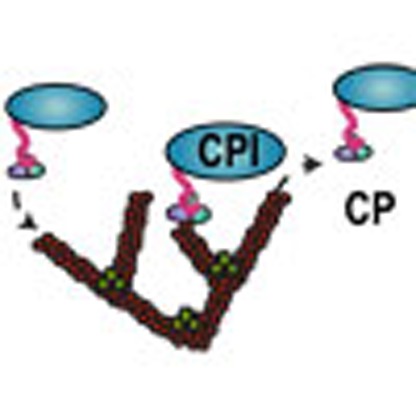

Actin filament assembly is the basis for the formation of many subcellular structures and for performance of various cell functions^1^. Actin filaments grow and shrink via addition and loss of actin subunits at their ends. In cells, free barbed ends are critical determinants of the spatial and temporal regulation of actin assembly. Heterodimeric actin-capping protein (CP) binds to and functionally caps free barbed ends, blocking their growth and shrinkage^2^, with subnanomolar binding affinity and a half-time for dissociation of ∼30 min^3^,^4^,^5^. CP is found in essentially all eukaryotic cells; its importance in regulating actin assembly and actin-based motility is likewise universal.

A diverse set of otherwise unrelated proteins contain a ∼30-residue motif, called CPI (capping protein interaction), that binds directly to CP and modulates its actin-capping activity^6^. CPI motifs are found in CARMILs, CKIP-1, CapZIP, CD2AP, the CD2AP homologue CIN85 and the WASH (Wiskott–Aldrich syndrome protein and SCAR homologue) subunit Fam21. For CARMIL1, CD2AP and CKIP-1, the CPI motif is necessary for actin-based cellular functions, based on the failure of mutations in the CPI motif to rescue knockdown or overexpression actin phenotypes^7^,^8^,^9^.

The traditional view of CP function in cells has been that CP is active and freely diffusible in the cytoplasm^10^; CP stochastically encounters and caps free barbed ends to terminate filament elongation^2^,^11^,^12^. This view is supported by synthetic studies with purified proteins in which capping barbed ends by diffusion is sufficient to promote actin-based motility^13^. In addition, mathematical models of actin-based motility in cells generally use the simplifying assumption that CP is a diffusible active capper, sufficient for activity on its own^14^,^15^.

On the other hand, models involving the regulation of CP in cells have been proposed. For example, uncapping of barbed ends capped by CP was proposed to generate free barbed ends during platelet activation, based on the observation that phosphatidylinositol 4,5-bisphosphate (PIP_2_) releases gelsolin and CP from barbed ends^16^. In addition, free barbed ends induced by Cdc42 in neutrophils are protected from CP^17^, and formins and enabled/vasodilator-stimulated phosphoprotein (Ena-VASP) proteins protect barbed ends from CP^6^.

More recently, discoveries of CPI-motif proteins have raised the possibility that CP might be targeted to specific cellular locations^18^, and have its capping activity decreased or even reversed (uncapping)^19^, to achieve proper actin filament dynamics. In support of this view, depletion of CARMIL1 and CD2AP was found to decrease localization of CP to the plasma membrane (PM)^7^,^8^. In addition, the protein V-1/myotrophin is known to inhibit CP *in vitro*^2^,^20^,^21^, and the CPI-motif protein CARMIL1 has been proposed to be part of a regulatory cycle promoting the release of CP from sequestration by V-1 (ref. 22).

Fundamental questions exist regarding the physiological function of the interaction of CPI-motif proteins with CP in cells. Do CPI-motif proteins decrease the actin-capping activity of CP at a given location or time, in order to limit or even reverse the capping of barbed ends? Alternatively, do CPI-motif proteins target active CP to sites where barbed ends need to be capped? Here, we tested the hypothesis that an interaction with a CPI-motif protein is required for CP localization and function by examining the cellular activities of CP mutants that are unable to bind to the CPI motif, but that retain full ability to bind and cap barbed ends. We found that these CP mutants do not function in cells, producing actin phenotypes identical to loss of CP. The mutants failed to rescue RNA interference-induced knockdown of CP, and the mutants had a dominant-negative effect in cells expressing endogenous CP.

## Results

### CP mutants defective in binding to CPI-motif proteins

To understand the role of CP regulation by CPI-motif proteins in cells, we created mutant forms of CP defective in binding the CPI motif, originally defined as LxHxTxxRPK(6X)P by Bruck *et al*.^23^. The CPI motif makes extensive and important close contacts with the CPβ subunit of the CP heterodimer^21^,^24^,^25^. For CPβ, the residues Arg15 and Tyr79 make close contact with critical residues of the CPI motif in co-crystal structures^24^. Accordingly, we changed these residues to Ala. The single mutants are R15A and Y79A, and the double mutant is R15A/Y79A (plasmids in Table 1). We predicted that these CP mutants would be deficient in binding to all CPI-motif proteins.

**Figure 1:**
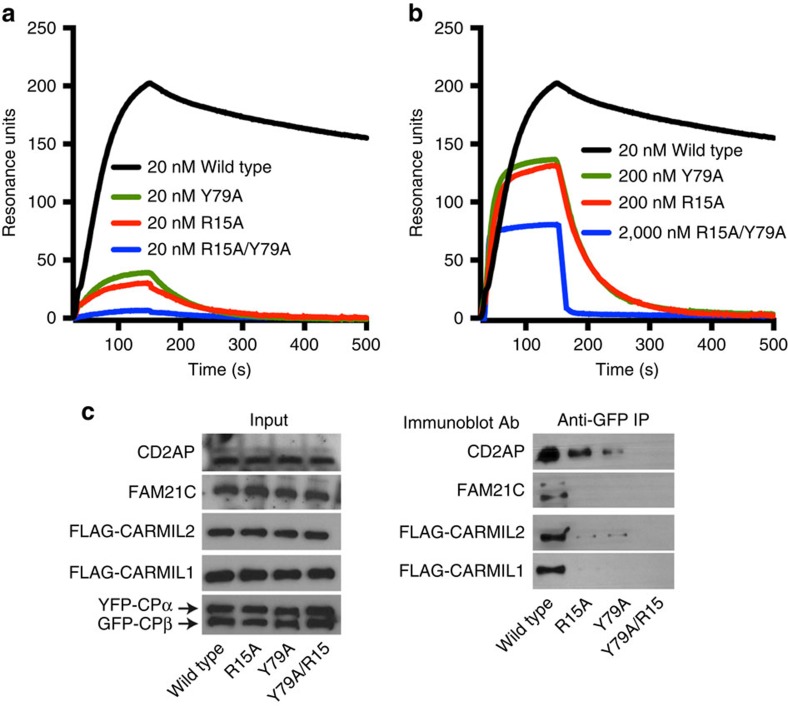
CPI-motif binding-site mutations impair CPI–CP interaction. (**a**) Interaction of purified CP and CARMIL-CBR, assayed by SPR (surface plasmon resonance). The SPR chip contains GST fused to CBR of human CARMIL1. CP at 20 nM was flowed over the chip. Black: wild-type CP, green: Y79A, red: R15A, blue: R15A/Y79A. One representative experiment, among four, is shown. (**b**) SPR traces as in **a**, with concentrations of CP as follows: wild-type 20 nM, R15A 200 nM, Y79A 200 nM, R15A/Y79A 2,000 nM. One representative experiment, among four, is shown. (**c**) Impaired association of CP mutants with CPI-motif proteins in cells. HT1080 cells expressed CP mutants tested in **a** and **b**. Cells were co-transfected with YFP-CPα1 and either GFP-CPβ2, GFP-CPβ2 R15A, GFP-CPβ2 Y79A or GFP-CPβ2 R15A/Y79A. CP was immunoprecipitated from whole-cell lysates with anti-GFP, and precipitates were probed with antibodies to endogenous CD2AP and Fam21C. For CARMIL1 and CARMIL2, endogenous levels were low, so cells were co-transfected with FLAG-tagged expression constructs, and IPs were probed with anti-FLAG.

We measured binding constants for the interaction of CP mutants with the capping protein-binding region (CBR) fragment of CARMIL1, which includes a CPI motif, using purified proteins and surface plasmon resonance (SPR). Representative SPR traces are shown in Fig. 1, and rate and binding constants based on complete data sets are listed in Table 2. SPR traces reveal that the binding affinity for each single mutant is less than that of wild-type CP by a factor of >10 and that the binding affinity for the double mutant is less by a factor of >100 (Fig. 1a,b). To calculate binding and rate constants, we fit complete time-course data for all the curves to a single-site model with 1:1 stoichiometry (Table 2). The kinetic modelling yielded independent values for association and dissociation rate constants, *k*_+_ and *k*_−_, which were used to calculate the binding constant, *K*_d_. The results for wild-type CP were similar to those from previous studies, with a *K*_d_<1 nM^5^. The *K*_d_ for each of the single mutants was ∼140 nM, with ∼10-fold decreases in *k*_+_ and ∼20-fold increases in *k*_−_. For the double mutant, the *k*_−_ was increased further, by a factor of 5, and *k*_+_ could not be determined because the extent of binding was too low. Thus, we estimate a lower limit for the *K*_d_ of >100 μM for the double mutant.

We tested the ability of four different CPI-motif proteins—CARMIL1, CARMIL2, CD2AP and FAM21C—to bind to the CP mutants in cells by immunoprecipitation (Fig. 1c). Interactions of all four proteins with the R15A/Y79A mutant were severely decreased, to near zero, based on the amounts of co-precipitated protein. For the single mutants, levels of co-precipitated proteins were also decreased, to very low or undetectable levels (Fig. 1c).

### Biochemical properties of CP mutants

**Figure 2:**
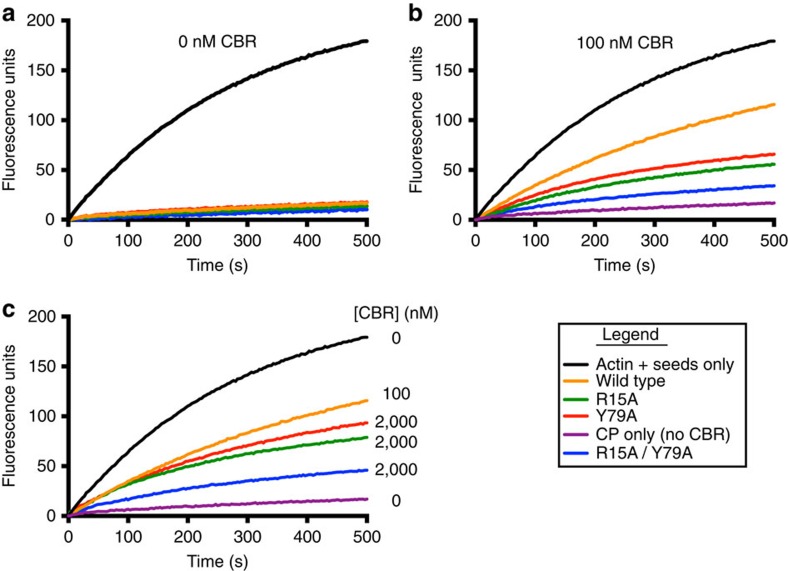
CP mutants cap actin normally but resist inhibition by the CPI motif. (**a**) Actin-capping activity is not affected by the CP mutations. Actin polymerization from barbed ends, revealed by pyrene–actin fluorescence versus time. CP (10 nM), wild-type and mutants, were added. Curve colours as follows: control without CP (black), wild-type CP (orange), CP R15A mutant (green), CP Y79A mutant (red) and CP R15A/Y79A mutant (blue). (**b**) Inhibition of CP by the CBR fragment of CARMIL1. Experiment as in **a**, with addition of 100 nM CBR at *t*=0. Colours as in **a**, with the addition of control with wild-type CP and no CBR (purple). (**c**) Similar to **b**, with higher concentration (2,000 nM) of CBR for CP R15A, Y79A and R15A/Y79A. The black, orange and purple curves are the same in **b** and **c**. For all panels, one experiment is shown, representative of three.

The rationale for our approach required that the CP mutants bind and cap actin filaments normally, which we expected based on the location of the mutated residues in the structure^24^. Indeed, we tested the actin-capping activity of the CP mutants over a range of concentrations and found them to be essentially identical to that of wild-type CP (Fig. 2a; Supplementary Fig. 1A–D).

We expected that decreased physical binding of the CP mutants to CPI motifs, as detected by SPR and co-immunoprecipitation, would be accompanied by decreased functional ability of CPI-motif proteins to inhibit the actin-capping activity of the CP mutants. We measured inhibition of capping activity for each CP mutant using CBR, a CPI-motif-containing fragment of CARMIL1 (Fig. 2b,c). As expected, the effect of CBR on CP mutants was much less than its effect on wild-type CP. The single-CP mutants were only partially inhibited by CBR, and the double-CP mutant showed only minimal inhibition, consistent with the physical binding assays. In one experimental design, CP and CBR concentrations were kept constant (Fig. 2b); in another design, CBR concentration was varied (Fig. 2c). Overall and most important, the double-CP mutant exhibited nearly undetectable interaction with CPI-motif proteins, in physical and functional assays.

We characterized the R15A/Y79A mutant further by testing interaction with the other known inhibitors of CP — PIP_2_ and V-1. V-1 competitively inhibits actin capping by binding to CP at a site that overlaps with the actin-binding site^26^. PIP_2_ also binds to and inhibits CP^27^,^28^. In actin-capping assays with purified proteins, addition of V-1 and PIP_2_ had similar inhibitory effects on wild-type CP and the R15A/Y79A mutant (Supplementary Fig. 2; Supplementary Fig. 3).

### Localization of CP mutants in cells

We tested the hypothesis that interaction with a CPI-motif protein is required for the localization of CP, as opposed to the simple alternative that CP freely diffuses to encounter and bind free barbed ends. The CPI-motif proteins CD2AP and CARMIL1 have been found to co-localize with CP in the actin-rich cell cortex^7^,^8^,^29^. In those studies, mutation of the CPI motif led to loss of CP localization at the cortex, but the mutations also led to loss of F-actin accumulation, leaving open the possibility that CP binding and accumulation depended on F-actin. In other studies, CP localized to macropinosomes and vesicular compartments in association with the CPI-motif protein Fam21, a component of the WASH complex^30^. Loss of Fam21 led to loss of CP and WASH co-localization at vesicle membranes; instead, CP was associated with abnormal F-actin streams emanating from vesicles, while WASH remained at the membrane^31^. These findings support the hypothesis that interaction with a CPI motif is required to localize CP at cellular membranes, rather than its passive diffusion and binding to actin filament barbed ends.

**Figure 3:**
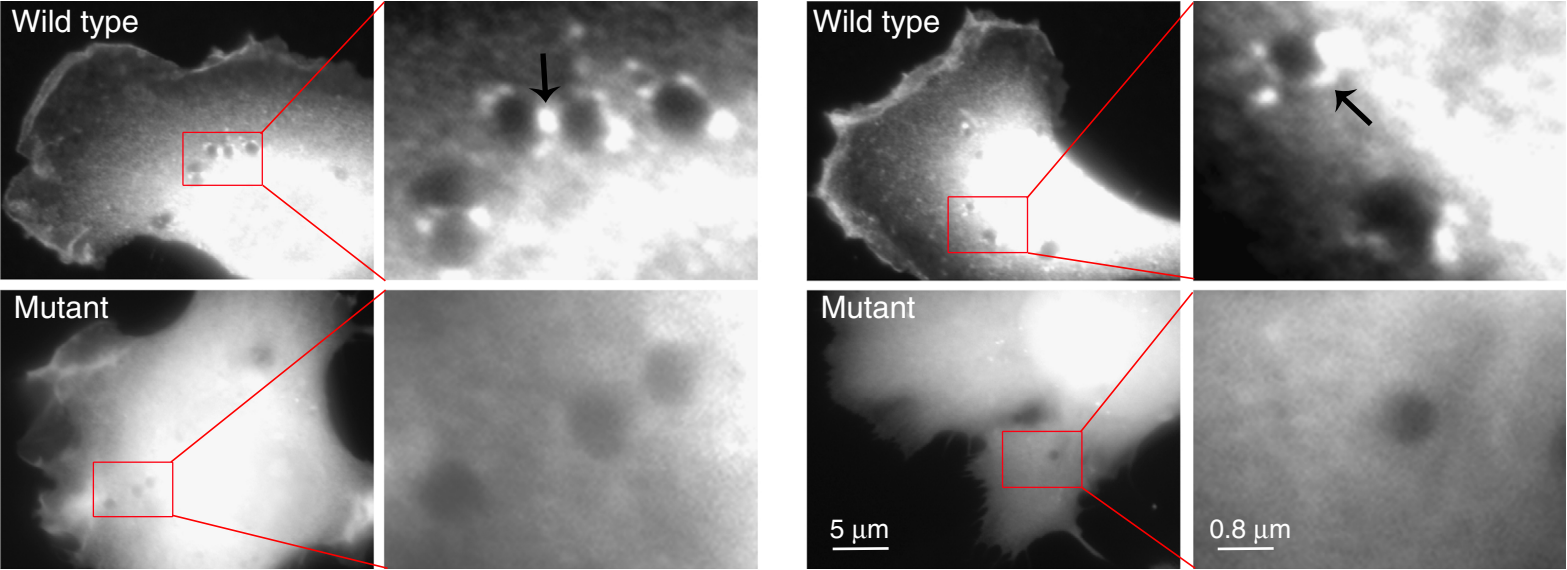
CP localization depends on the ability to bind CPI-motif proteins. GFP-tagged fusions of wild-type CPβ2 or the R15A/Y79A mutant were expressed in cells at low levels. Cells were fixed and immunostained with anti-GFP. Representative images are shown; *n*=27 cells. Red rectangles indicate the region of the cell magnified in the adjacent inset. Arrows indicate puncta of CP. The expression levels in this experiment were far lower than the levels used to induce changes in cell shape and actin distribution, described later.

To test this hypothesis, we localized green fluorescent protein (GFP)-tagged versions of wild-type CPβ2 (ref. 30) and double-mutant CP (R15A/Y79A β2) (Fig. 3). GFP–CP fusions were expressed in HT1080 cells at very low levels, so that neither wild-type nor mutant produced any observable effect on the morphology or actin cytoskeleton of the cells. Wild-type CP was enriched at the leading edge of cells and concentrated in puncta associated with macropinosomes and vesicular structures (Fig. 3, arrows). In contrast, the R15A/Y79A mutant remained diffuse and failed to localize to the leading edge of cells and to macropinosomes, in all of the 37 cells analysed (Fig. 3; Supplementary Fig. 4)

We quantified PM localization by calculating the PM index^32^, which accounts for volume effects by normalizing the membrane-associated signal to that of a cytoplasmic marker. The PM index of GFP was near zero (0.06), as expected for a freely diffusing protein. The value for wild-type CP was 1.28, and the value for the R15A/Y79A mutant was 0.12 (Supplementary Fig. 4). Because the R15A/Y79A mutant binds barbed ends normally, we conclude that localization of CP in cells requires binding interaction with a CPI-motif protein, not to barbed ends of actin filaments.

### Function of CP mutants in cells

To assess the physiological importance of the CP–CPI interaction, we asked whether and how CP mutants function in cells, focusing on actin assembly and dynamics. We reasoned that if CPI motifs target or recruit CP to certain locations and if recruitment is necessary for function, then the phenotype of cells expressing CP mutants should phenocopy the loss of CP. Alternatively, if the role of CPI motifs is to inhibit CP and prevent capping or promote uncapping, then CP mutants unable to bind CPI motifs should cap constitutively, and their cellular phenotype should resemble the effects of increased levels of CP and actin capping.

**Figure 4:**
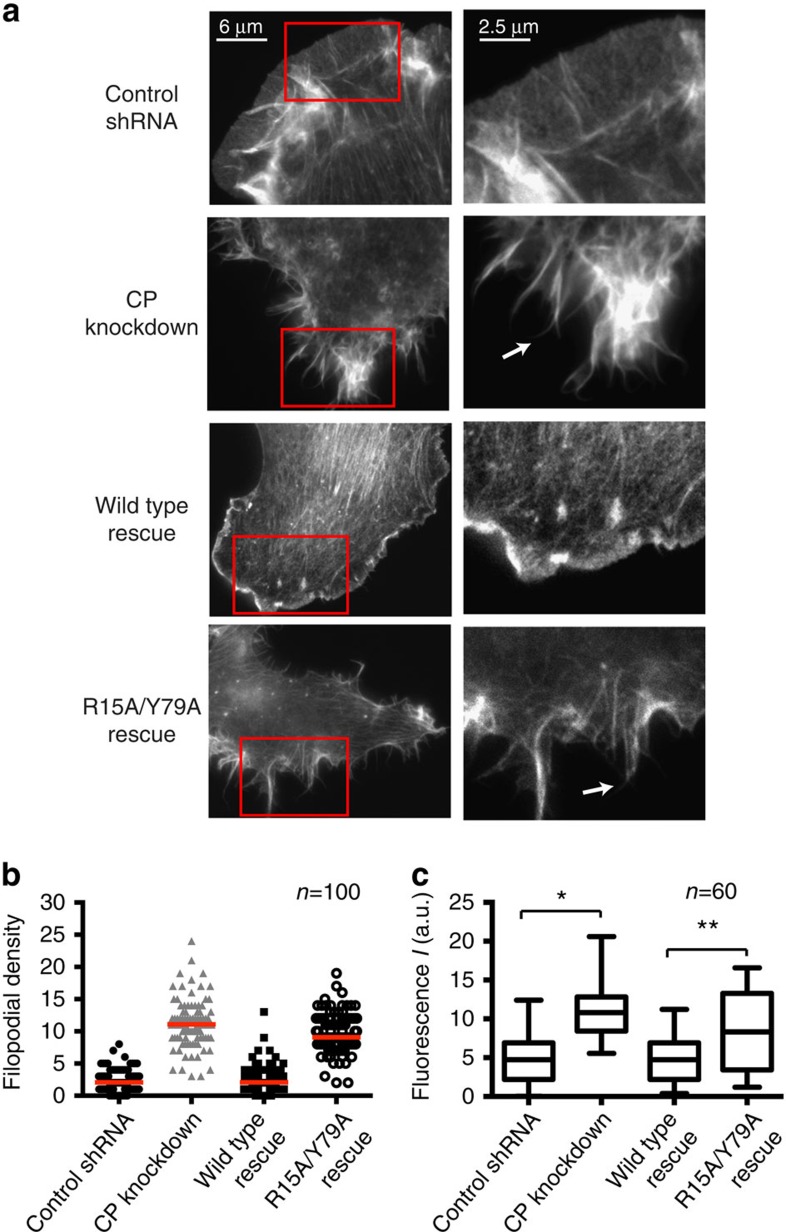
CP mutants fail to rescue CP-depletion phenotypes. (**a**) Images of CP-depleted cells expressing wild-type CP or R15A/Y79A mutant, stained with fluorescent phalloidin. Arrows indicate filopodia. Red boxes in left panels are magnified on the right. (**b**) Quantification of filopodia density. The *y* axis is the number of filopodia per 10 μm of cell perimeter; *n*=100 cells. (**c**) Quantification of whole-cell phalloidin staining density from (**a**). The difference between control shRNA and CP knockdown is significant (*P<0.01); the difference between mutant and wild-type rescue is also significant (**P<0.02).

To test these hypotheses, we expressed the CP β-subunit mutants in HT1080 cells depleted of endogenous CP by targeting the β-subunit with short hairpin RNA (shRNA) (Fig. 4). CP depletion led to a loss of lamellipodia and an increase in filopodia, consistent with previous results in mouse B16 cells^33^ and *Dictyostelium*^34^. Expression of shRNA-resistant wild-type CP restored the actin phenotype to that of control cells; lamellipodia appeared and filopodia were lost. In contrast, expression of shRNA-resistant R15A/Y79A mutant did not provide rescue; cells lost lamellipodia and gained filopodia, similar to CP-depleted cells. Representative images are shown in Fig. 4a, and quantitative analysis of filopodia number, scored by a blinded observer, is plotted in Fig. 4b. In addition, CP depletion caused an increase in the total level of F-actin per cell, based on fluorescent phalloidin (Fig. 4c), consistent with previous reports^34^,^35^. This phenotype was also rescued by expression of wild-type CP, but not the R15A/Y79A mutant (Fig. 4c). The single mutants, R15A or Y79A, produced results similar to wild type (not shown). The levels of expressed double- and single-mutant forms of CP were the same as or slightly greater than the level of endogenous wild-type CP by immunoblot (Supplementary Fig. 5).

**Figure 5:**
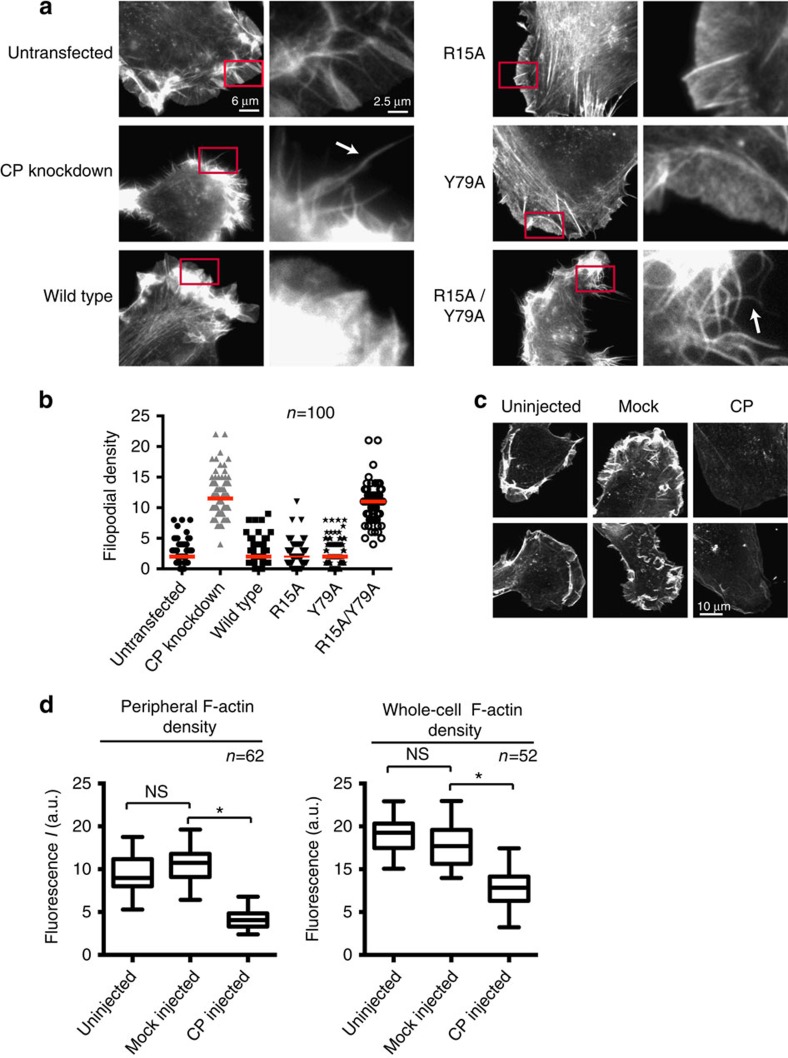
Overexpression of CP R15A/Y79A mutant mimics depletion of CP. (**a**) Cells were transfected with the indicated constructs and stained with fluorescent phalloidin. Arrows indicate filopodial protrusions. Red boxes in left panels are magnified to the right. (**b**) Quantification of filopodia density. The *y* axis is the number of filopodia per 10 μm of cell perimeter; *n*=100 cells. (**c**) Increased cellular CP did not affect F-actin organization. Cells were microinjected with a mixture of purified wild-type CP (78.5 μM) and a fixable rhodamine-dextran marker. Cells were stained with fluorescent phalloidin. Mock-injected cells received only the rhodamine-dextran marker. Maximum-intensity projections of confocal images are shown. (**d**) Quantification of whole-cell and cortical phalloidin staining densities from **c**. The difference between CP-injected and mock-injected cells is statistically significant (*P<0.01), whereas the difference between mock-injected and uninjected cells is not. NS, not significant.

To test the function of the CP mutants further, we expressed them in cells where endogenous CP was not depleted (Fig. 5a,b). Mutant or wild-type forms of CPβ were transiently expressed, along with the wild-type CPα subunit. Expression of wild-type CP or the two CP mutants with single amino-acid changes, R15A or Y79A, had small effects on cell morphology (Fig. 5a) and on the quantitative scores of lamellipodia and filopodia (Fig. 5b). In contrast, expression of the R15A/Y79A mutant caused large effects, with decreased lamellipodia and increased filopodia, closely resembling the effects of depleting CP (Fig. 5b). Together the results show that a mutant form of CP defective in binding to CPI-motif proteins has a dominant-negative effect on cells, which argues that the mutant β-subunits fold and interact with the α-subunit in a normal manner. On the basis of these results, we conclude that CP is required to bind to a CPI-motif protein to function in cells.

### Increasing cellular (CP) decreases cellular F-actin levels

To test the model in which CPI-motif proteins only inhibit CP, without spatial targeting of capping activity, we sought additional evidence that the expressed CP mutant proteins did not provide a gain of CP function in cells, and thereby alter cellular actin structures. In previous studies with *Dictyostelium*, the effects of overexpressing CP on F-actin structures was clearly distinct from that of depleting CP^34^. To increase the level of CP in HT1080 cells, we microinjected purified wild-type CP, at the highest concentration possible, into the cytoplasm of cells with a microneedle (Fig. 5c). Cells, which typically have 1–2 μM cytoplasmic CP^17^,^36^, were injected with purified CP, producing a four- to eightfold increase in cellular CP concentration.

CP-injected cells displayed a distinct lack of ruffling at the periphery when compared with uninjected or mock-injected cells (Fig. 5c). The number of filopodia was not increased, in striking contrast to the loss of CP. In addition, CP-injected cells exhibited decreased F-actin density, both in the lamellipodial region at the cell edge, and globally, as quantified from the intensity of fluorescent phalloidin staining (Fig. 5d). Thus, the effects of overexpressing and depleting CP in HT1080 cells differed, as observed in *Dictyostelium*^34^. These results demonstrate that the effects of the CP mutant proteins on cellular actin structures do not result solely from elevated actin-capping activity. We conclude that the CPI motif acts to selectively target CP to specific actin networks, where it promotes uncapping of capped actin filaments or provides a decreased level of capping activity tuned to the dynamics of actin assembly.

### Loss of CP function resembles loss of Arp2/3 function

**Figure 6:**
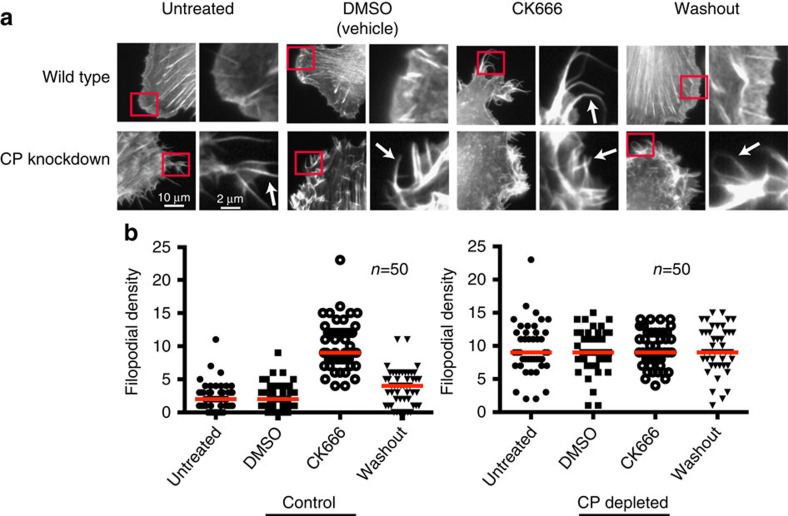
Effects of inhibition of Arp2/3 complex resemble loss of CP function. **a**) Cells were treated with the Arp2/3 complex inhibitor CK-666 in dimethyl sulfoxide (DMSO) for 3 h, then stained with fluorescent phalloidin. Negative controls included no treatment and vehicle (DMSO). In the washout sample, CK-666 was removed by washing with growth media, five times over 30 min. As a control, the inactive compound CK-689 produced no effect. Upper row: control cells. Lower row: cells treated with lentivirus-expressing CP shRNA targeting the CP β-subunit. Arrows indicate filopodia. Representative images from 20 cells are shown. (**b**) Quantification of normal lamellipodia (black) and increased filopodia (grey). The *y* axis is the number of cells; *n*=50.

In the dendritic nucleation model for actin assembly induced by Arp2/3 complex, CP has an essential role, capping barbed ends after they have grown for a short period of time. This view is supported by the observation that CP depletion leads to loss of the branching actin filament networks in lamellipodia^33^, as well as the finding that a barbed-end capper, such as CP, is a necessary component for synthetic reconstitution of Arp2/3-mediated actin-based motility^37^,^38^. Thus, we asked whether loss of function of CP in HT1080 cells resembles loss of function of the Arp2/3 complex by treating cells with the Arp2/3 inhibitor CK-666 (ref. 39). The effects on lamellipodial F-actin were similar to those observed when CP was depleted (Fig. 6). Furthermore, as a test for functional overlap between CP and the Arp2/3 complex, we treated CP-depleted cells with CK-666. No additional effects on F-actin organization were observed, indicating that CP and the Arp2/3 complex are both necessary for lamellipodia formation, as predicted by the dendritic nucleation model^12^.

## Discussion

We discovered that CP requires an interaction with a CPI-motif protein for its function in cells. This conclusion contradicts the idea that CP randomly diffuses about the cytosol, stochastically colliding with free barbed ends and capping them^2^. This view of CP function has been widely held and applied since its discovery and initial characterization 35 years ago^10^; the dendritic nucleation model for Arp2/3-based actin assembly incorporates this view implicitly^12^. Moreover, this view is an explicit assumption of many physical and mathematical models for actin assembly and actin-based motility^14^,^15^,^40^.

Our conclusion is based on the observation that point mutations of CP, which prevent its binding to a CPI-motif protein, but which retain normal filament capping activity, mimic the loss of CP function in cells. The CP mutations caused loss of binding to CPI-motif proteins in biochemical assays with purified proteins, and they abrogated interactions of CPI-motif proteins with CP in cells, based on pulldowns from whole-cell lysates. Finally, we found that the CPI-binding mutant CPs localized diffusely in the cytoplasm and did not accumulate at sites of dynamic actin assembly as wild-type CP did.

**Figure 7:**
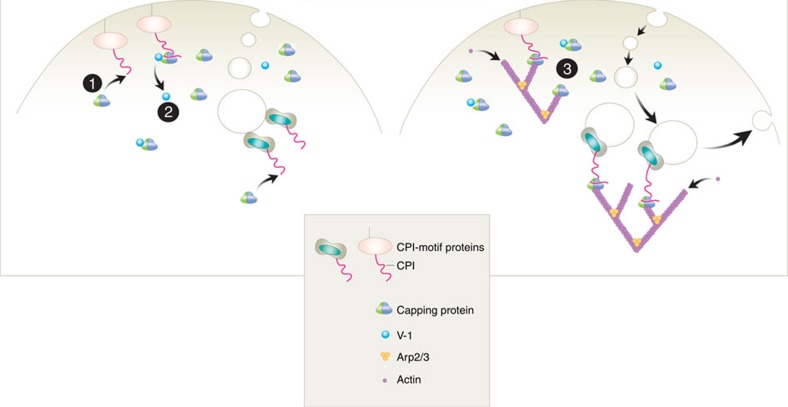
CP localization and function requires interaction with CPI-motif proteins. CPI-motif proteins facilitate the recruitment of free cytosolic CP to biological membranes via the CPI motif (1). The CPI–CP interaction accelerates the dissociation of V-1 bound to CP (2). CPI–CP complex caps the barbed end of growing actin filaments near the membrane and promotes dendritic actin network growth and dynamics (3).

Together our findings suggest new models for regulation of actin assembly in cells by CP. We envision two important functions, not exclusive of one another, that result from the essential interaction of CP with a CPI-motif protein. First, a CPI-motif protein may target CP, and therefore actin-capping activity, to specific locations in the cell, such as membranes (Fig. 7). Second, the CPI motif/CP interaction may tune the barbed-end capping activity of CP to an optimal range, appropriate for the temporal regulation of actin dynamics in cells, by increasing the rate at which CP dissociates from capped barbed ends. For actin filaments capped by CP, CPI-motif proteins may uncap the barbed ends.

CPI motifs are found in a diverse set of otherwise unrelated proteins^6^. Most CPI-motif proteins bind to PMs and intracellular membranes via distinct domains, suggesting that they serve as platforms for spatiotemporal regulation of actin assembly dynamics. Previous studies of two CPI-motif proteins, CD2AP and CARMIL1, demonstrate their ability to both target CP and tune its actin-capping activity^7^,^8^. CPI-motif proteins vary in their affinity for CP and in the extent to which they modulate capping activity. One set of CPI-motif proteins, the CARMIL family, have a second motif that also interacts with CP, termed the CARMIL-specific interaction motif^24^. This second motif appears to enhance the binding affinity and regulatory effects of CP. The range of capping activities exhibited by various CPI–CP complexes, together with independent regulation of these interactions by signalling proteins, suggests an underappreciated mechanism for local regulation of actin polymerization induced by the Arp2/3 complex and formins at membranes.

Previous studies provided evidence for the existence of CP inhibitors in cells. The apparent affinity of CP for barbed ends in cells is ∼100-fold weaker than the calculated subnanomolar affinity for actin filaments observed *in vitro*^34^. The time for dissociation of CP from barbed ends of actin filaments *in vitro* is ∼500-fold longer than the dissociation rates in cells observed by single-molecule analysis^4^,^41^. The ∼50-fold decreased affinity of the CP–CPI capping complex for the barbed end *in vitro* suggested one possible explanation for this discrepancy^18^, and the existence of a ternary CP–CPI–actin complex, implied by this model, was demonstrated clearly *in vitro*^22^,^42^. Indeed, our results here argue that such a complex is physiologically relevant in cells (Fig. 7).

The protein V-1, also known as myotrophin, binds to CP at its actin-binding site, serving as a competitive inhibitor of capping *in vitro.* If V-1 also functions in this manner in cells, then CPI-motif proteins may form part of a regulatory cycle for CP that counteracts and relieves inhibition by V-1 (ref. 22). In such a model, proposed by Hammer *et al*., the allosteric effects of a CPI-motif protein, such as CARMIL1, would release CP from inhibition by V-1, creating a CP–CPI complex with physiological capping activity. Our results here are consistent in principle with such a model. The role of V-1 in cells with respect to actin remains relatively uncertain, especially in light of the known interaction of V-1 with NF-kB signalling pathways^43^.

Our results, combined with previous studies on regulation of CP by a variety of proteins and lipids, indicate that we have much to learn about the dynamics of actin assembly in cells. The physiological significance of individual regulatory interactions and mechanisms for combining the actions of individual regulators are important open questions.

## Methods

### Antibodies and reagents

Reagents and materials were from Fisher Scientific (Pittsburgh, PA) or Sigma-Aldrich (St Louis, MO), unless stated otherwise. Antibody to CP was mouse monoclonal antibody clone 2A3 for immunoblots^4^. Other antibodies and sources were as follows: Fam21C (rabbit pAb) from Millipore (Billerica, MA), anti-CD2AP (rabbit pAb) was a kind gift from Dr Andrey Shaw of Washington University, goat anti-Dap12 from Santa Cruz (Dallas, TX), anti-GFP (rabbit pAb) and rabbit anti-GST antibody from Abcam (Cambridge, MA) and anti-FLAG (mouse monoclonal antibody M2) from Sigma-Aldrich. Dynabeads, Protein G, HRP- and Alexa-conjugated secondary antibodies and Alexa 488- and Alexa 568-conjugated phalloidin were from Life Technologies (Carlsbad, CA). Arp2/3 complex inhibitors CK-666 and CK-869 (ref. 39), along with the control compound CK-689, were purchased from Sigma-Aldrich. pERFPC-1 and pEGFPC-1 were obtained from Clontech (Mountain View, CA). PIP_2_ was obtained from Avanti Polar Lipids (Alabaster, AL).

### Protein expression and purification

Plasmids used in this study are listed in Table 1. CP mutants were generated by site-directed mutagenesis (QuikChange, Stratagene Agilent, La Jolla, CA) of the His-tagged expression plasmid. Arginine 15 and tyrosine 79 of mouse CPβ2 (GenBank FJ692320.1) were both changed to alanine to produce two single mutants R15A and Y79A and one double mutant R15A/Y79A.

His-tagged CP proteins were expressed in *Escherichia coli* BL21 (DE3, pRIL). They were purified as described^24^, with gel filtration chromatography on Sephacryl S-200 HR (GE Healthcare) in 20 mM Tris-HCl pH 7.4, 50 mM KCl, 0.5 mM Tris(2-carboxyethyl)phosphine (TCEP) and 1 mM NaN_3_. Proteins were snap-frozen in liquid nitrogen and stored at −70 °C.

Glutathione *S*-transferase (GST)-tagged CBR fragment of human CARMIL1a (GenBank FJ009082), residues Glu964 to Ser1078 was expressed in *E. coli* BL21 (DE3, pRIL), affinity-purified on Glutathione Sepharose Fast-Flow (GE Healthcare), followed by chromatography on DEAE Sepharose Fast-Flow (GE Healthcare). Purified GST-CBR was exchanged into 20 mM Tris-HCl pH 7.4, 50 mM KCl, 0.5 mM dithiothreitol, 1 mM NaN_3_, snap-frozen in liquid nitrogen and stored at −70 °C.

GST-tagged V-1 was expressed in *E. coli* BL21 Star (DE3) (Life Technologies), affinity-purified on Glutathione Sepharose Fast-Flow (GE Healthcare), chromatographed on Sephacryl S-200 HR (GE Healthcare) in 20 mM Tris-HCl pH 7.4, 50 mM KCl, 1 mM TCEP, 1 mM NaN_3_, snap-frozen in liquid nitrogen and stored at −70 °C.

### Actin polymerization assays and SPR

Pyrene–actin polymerization assays were performed as described^40^. Elongation rates were measured using time-based scans on a steady-state fluorometer (QuantaMaster, PTI, Edison, NJ) with excitation at 368 nm and emission at 386 nm. Actin monomer concentration was 1.5 μM with 5% pyrene labelling. The indicated concentrations of CP, GST-CBR, GST-V-1 and PIP_2_ were added at the start of the experiments. Pyrene–actin filament seeds were prepared as described^44^. Spectrin–actin seeds were prepared from a low-ionic-strength extract of bovine red blood cell ghosts as described^45^. Extraction of the ghost pellet was performed at 37 °C for 40 min followed by centrifugation at 110,000 *g*. The supernatant was collected and an equal volume of ethylene glycol was added for storage at −20 °C.

Binding kinetics were measured by SPR using a Reichert SR7000 Single Channel Surface Plasmon Resonance System at 25 °C. Rabbit anti-GST antibody was immobilized onto a carboxymethyldextran chip (Reichert Technologies, gold sensor slide, 500,000 Da) using carbodiimide coupling. Goat anti-Dap12 Ab was used as a negative control. GST-CBR was injected onto the chip containing the immobilized antibodies. Next, wild-type or mutant CP was injected at 25 μl min^−1^. The buffer for GST-CBR and CP was 20 mM Tris-HCl pH 7.4, 150 mM NaCl, 5 mM EDTA, 0.5 mM TCEP, 0.005% Tween-20 and 1 mM NaN_3_. Chip regeneration was performed with 3.5 M MgCl_2_, 20 mM MES pH 6.5.

CP mutants were injected over a range of concentrations, and kinetic rate constants were obtained using Scrubber V2.0c software (Biologic Software, Canberra, Australia). The dissociation phase was fit well by an exponential decay model for all mutants. Using the value for *k*_−_ from the dissociation phase, the association phase was fit well by a single-site two-component binding model, yielding a value for *k*_+_. *K*_d_ was calculated as the quotient of the rate constants. For the weak-binding R15A/Y79A double-mutant, fitting the association phase was not possible because the signal was too low. As part of the analysis, we considered a model including an additional step for mass transport, along with binding, which is sometimes necessary depending on the conditions and design of an SPR experiment. Adding the transport step did not improve the fit by a significant amount, so we did not use this model.

### Cell culture and lentiviral expression

Human HEK-293 cells and HT1080 cells (American Type Culture Collection, Manassas, VA) were cultured in DMEM (Life Technologies) supplemented with 10% fetal bovine serum (Sigma-Aldrich) in 5% CO_2_. Cells were transfected using Transit LT1 (Mirus, Madison, WI).

Dr Wei-Lih Lee made the expression construct pEYFP-CPα1 by fusing mouse CPα1 complementary DNA (GenBank U16740.1) to the C terminus of yellow fluorescent protein (YFP) in the PEYFP C-1 vector (Clontech). Site-directed mutagenesis was performed on pEGFP-CPβ2 (ref. 30) to prepare GFP-CPβ2 mutants.

To deplete endogenous CP, we infected cells with lentivirus-expressing shRNA in pLKO.1 (ref. 46). Infected cells were selected with puromycin and used for experiments at 72 h. The shRNA sequence was 5′-GCCTGGTAGAGGACATGGAAA-3′, which targets all isoforms of human CPβ, because the isoforms are produced by alternative splicing from a single gene, and this sequence is present in the mature messenger RNA of all splice variants. The shRNA construct was developed by the RNAi Consortium at the Broad Institute^47^ and purchased from the Children's Discovery Institute/Genome Sequencing Center at Washington University (St Louis, MO). A non-targeting shRNA sequence recommended for the library was used as a control (Sigma-Aldrich).

For knockdown/expression experiments, cells were first infected with lentivirus-expressing shRNA targeting CP. After 72 h, 10^6^ infected cells were transfected with 5 μg of YFP CPα1 and 5 μg of GFPβ2 wild-type, R15A, Y79A or R15A/Y79A, then fixed and stained after an additional 24 h. For overexpression, 10^6^ cells were transfected with 5 μg of YFP CPα1 and 5 μg of GFP–CPβ2 wild-type, R15A, Y79A or R15A/Y79A DNA and fixed after 48 h. Cells were positive for both YFP and GFP, based on imaging on a Zeiss 510 laser scanning confocal microscope with a Meta detector. For localization experiments, cells were transfected with 1 μg of CPβ2 wild-type or R15A/Y79A and fixed and stained with the indicated antibodies after 36 h.

### Microinjection and immunofluorescence

For microinjection, HT1080 cells were injected with a solution containing 5.1 mg ml^−1^ mouse CPα1β2 and 0.05 mg ml^−1^ fluorescent fixable dextran (Sigma-Aldrich) in injection buffer (10 mM potassium phosphate, pH 7.5, 75 mM KCl). Cells were fixed 15 min after injection, stained with Alexa 488-conjugated phalloidin and imaged with a × 63/1.4 numerical aperture (NA) objective on a Zeiss 510 laser scanning confocal microscope (Zeiss, Jena, Germany). Control cells were injected with fluorescent dextran only.

For immunofluorescence, HT1080 cells were plated for 3 h at 37 °C on glass coverslips previously coated with 15 μg ml^−1^ fibronectin (Sigma-Aldrich). Cells were fixed in 2% formaldehyde with 0.25% glutaraldehyde for 10 min at 37 °C and processed as described^33^. Immunostaining was performed with primary and secondary antibodies listed above at concentrations of 1 and 5 μg ml^−1^, respectively. Cells were imaged with × 100/1.4 NA and × 40/0.75 NA objectives on an Olympus IX70 inverted microscope (Olympus, Melville, NY) equipped with a cooled CCD camera (CoolSnap, Photometrics, Tucson, AZ), a Zeiss Axiovert 200 (Carl Zeiss AG, Oberkochen, Germany) with a × 100/1.3 NA objective and a Zeiss 780 (Carl Zeiss AG) with a × 63/1.5 NA objective.

Filopodia density was quantified by counting phalloidin-stained filopodia per 10 μm of cell perimeter. Fluorescence intensity in selected regions of interest was calculated using ImageJ^48^ and the following formula: integrated density−(mean background fluorescence × area of the region of interest).

To quantitate fluorescence of GFP-CP at the plasma membrane, we co-expressed RFP (red fluorescent protein) as a volume marker for the cytoplasm.

PM index, ((GFPm/RFPm)/(GFPc/RFPc))-1, was calculated as described^32^ with the following modifications. Using ImageJ, GFPm/RFPm was calculated by dividing the GFP image by the RFP image, then measuring fluorescence intensity along a 3-μm-wide band drawn around the cell cortex using the freehand-line tool. GFPc and RFPc denote average cytoplasmic fluorescence intensity and was calculated using the freehand-line tool in ImageJ to define an irregularly shaped region of the cytoplasm between the membrane and nucleus. Values for PM index are near 0 for cytoplasmic proteins and >1 for PM proteins. The statistical significance of the membrane indices was determined using the Student's *t*-test, with a two-tailed non-paired comparison.

### Co-immunoprecipitation and immunoblots

Anti-GFP was coupled to protein A Dynabeads at a concentration of 1 mg ml^−1^ (Life Technologies) according to the manufacturer's instructions. Cells were harvested 24 h after transfection in lysis buffer (PBS with 0.1% NP-40 and protease inhibitors aprotinin, bestatin, leupeptin, E-64, pepstatin A and phenylmethylsulphonyl fluoride). Cleared cell lysate was incubated with anti-GFP-coupled protein A Dynabeads for 3 h at 4 °C. Beads were washed five times with PBS. Precipitated proteins were eluted by boiling in SDS and analysed by SDS–polyacrylamide gel electrophoresis and immunoblots. Blots were developed with electrochemiluminescence (ECL; PerkinElmer-Cetus, Boston, MA) and exposed to autoradiography film.

### Arp2/3 complex inhibition

Cells were plated on fibronectin-coated coverslips and incubated with 100 μM of CK-666, CK-869 or CK-689, or the vehicle dimethyl sulfoxide for 3 h at 37 °C in 5% CO_2_. For washout assays, cells were washed with growth media five times for a total of 30 min. Cells were then fixed and stained with fluorescent phalloidin.

## Additional information

**How to cite this article:** Edwards, M. *et al*. CPI motif interaction is necessary for capping protein function in cells. *Nat. Commun.* 6:8415 doi: 10.1038/ncomms9415 (2015).

## Supplementary Material

Supplementary Information Supplementary Figures 1-5

## Figures and Tables

**Table 1:** Plasmids used in this study.

**pBJ #**	**Name**	**Description**
1678	Control shRNA	Control shRNA
2377	CP shRNA	shRNA for targeting CP
2086	pHR'8.2 ΔR	Lentiviral packaging plasmid
2087	pCMV-VSV-G	Lentiviral packaging plasmid
1841	GST-CARMIL1 CBR115aa	Bacterial expression of GST-CBR
2041	His-CPα1β2	Bacterial expression of wild-type CP
2302	His-CPα1β2 R15A	Bacterial expression of R15A CP
2303	His-CPα1β2 Y79A	Bacterial expression of Y79A CP
2304	His-CPα1β2 R15A/Y7A	Bacterial expression of R15A/Y79A CP
2438	GST-V-1	Bacterial expression of GST-V-1
2358	YFP-CPα1	Expression of YFP-CPα1 in mammalian cells
2359	GFP-CPβ2	Expression of GFP-CPβ2 in mammalian cells
2361	GFP-CPβ2 R15A	Expression of GFP-CPβ2 R15A in mammalian cells
2362	GFP-CPβ2 Y79A	Expression of GFP-CPβ2 Y79A in mammalian cells
2363	GFP-CPβ2 R15A/Y79A	Expression of GFP-CPβ2 R15A/Y79A in mammalian cells

CBR, capping protein-binding region; CP, capping protein; GFP, green fluorescent protein; GST, glutathione *S*-transferase; shRNA, short hairpin RNA.

**Table 2:** Rate and binding constants for CP mutants.

**CP species**	**[Analyte] (nM)**	***k***_**+**_ **(M**^**−1**^ **s**^**−1**^**) (× 10**^**5**^)	***k***_**−**_ **(s**^**−1**^**) (× 10**^**−3**^)	***K***_**d**_ **(nM)**
Wild type	20	10.4±1.5	0.60±0.01	0.60±0.11
R15A	200	1.26±0.10	20.6±1.7	165±25
Y79A	200	1.6±0.2	19.8±0.3	125±16
R15A/Y79A	2,000	ND (<0.01)	113±26	ND (>100 μM)

CP, capping protein; ND, not determined; SPR, surface plasmon resonance.

Determined by SPR, based on fitting to curves as described in Methods. A term for mass transport did not improve the fit by a statistically significance amount. The dissociation rate constant *k*_−_ was determined first, from the dissociation phase. With *k*_−_ fixed, the association rate constant *k*_+_ was then determined from the association phase. *K*_d_ is the quotient of *k*_−_/*k*_+._ ND—not determined because of weak signal and high error due to low binding.
