# Supplementary material for: CPI motif interaction is necessary for capping protein function in cells
Source: Nat Commun. 2015 Sep 28;6:8415. doi: 10.1038/ncomms9415 (PMC4598739; doi:10.1038/ncomms9415)
Supplement: Supplementary Information — Supplementary Figures 1-5 [file ncomms9415-s1.pdf]

Supplementary Information

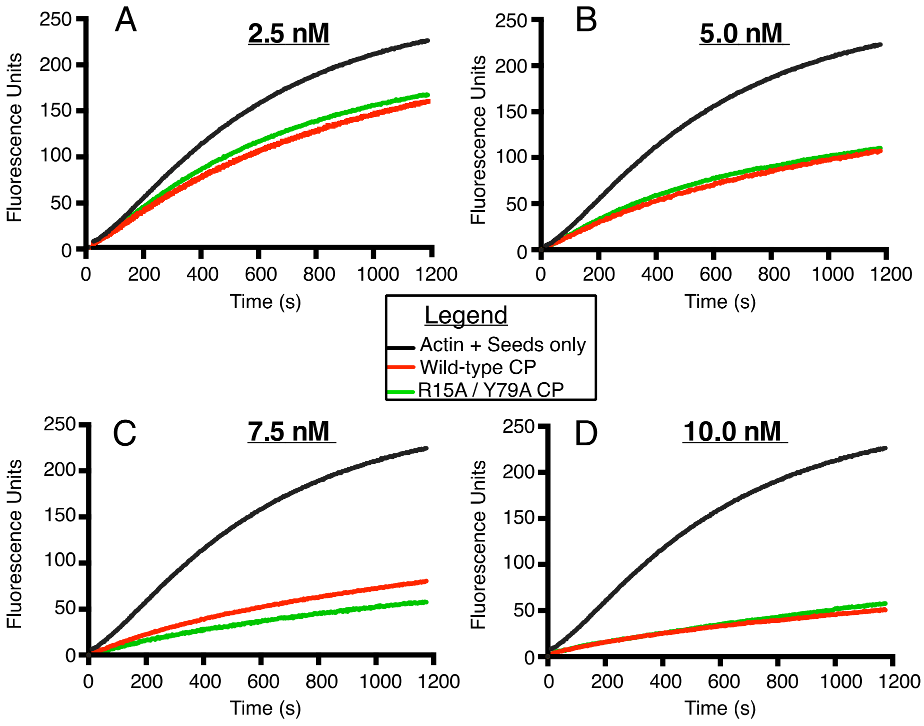

Supplementary Figure 1. CP R15A/Y79A mutant Caps Actin Normally. Actin capping activity is not affected by the CP mutations. Actin polymerization from barbed ends, with pyrene-actin fluorescence plotted vs. time. Experiments were performed with the indicated concentrations of wild-type CP (red curves) and R15A/Y79A mutant (green curves). CP concentrations are indicated on the panels.

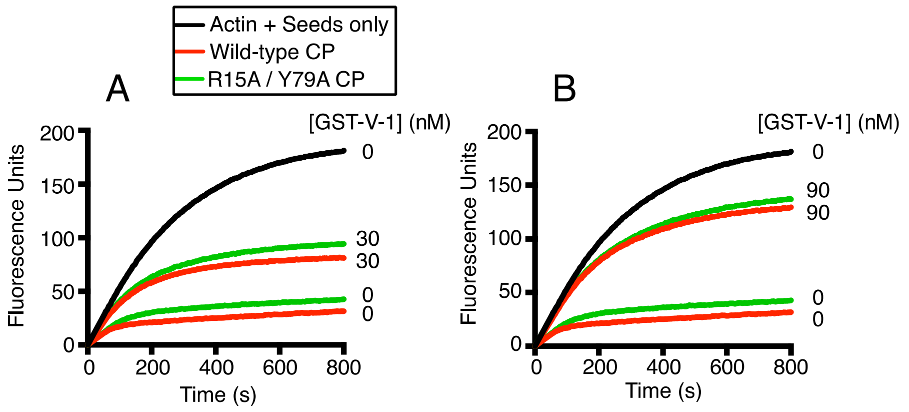

Supplementary Figure 2. Inhibition of CP by V-1 in Actin Capping Assays. V-1 inhibits wild-type CP and R15A/Y79A mutant CP to very similar, nearly identical,

degrees. Actin polymerization from barbed ends, measured by pyrene-actin fluorescence, is plotted vs. time. GST V-1 was added at the beginning of the assays at the concentrations indicated.

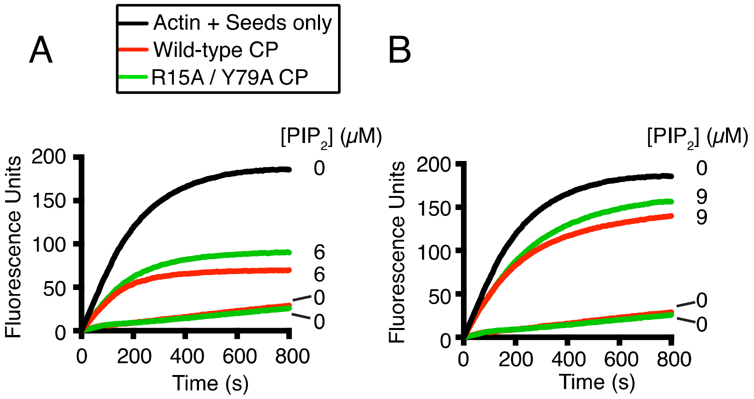

Supplementary Figure 3. Inhibition of CP by PIP_2 in Actin Capping Assays. PIP_2 inhibits wild-type CP and R15A/Y79A mutant CP to very similar, nearly identical, degrees. Actin polymerization from barbed ends, measured by pyrene-actin fluorescence, is plotted vs. time. PIP_2 was added at the beginning of the assays at the concentrations indicated.

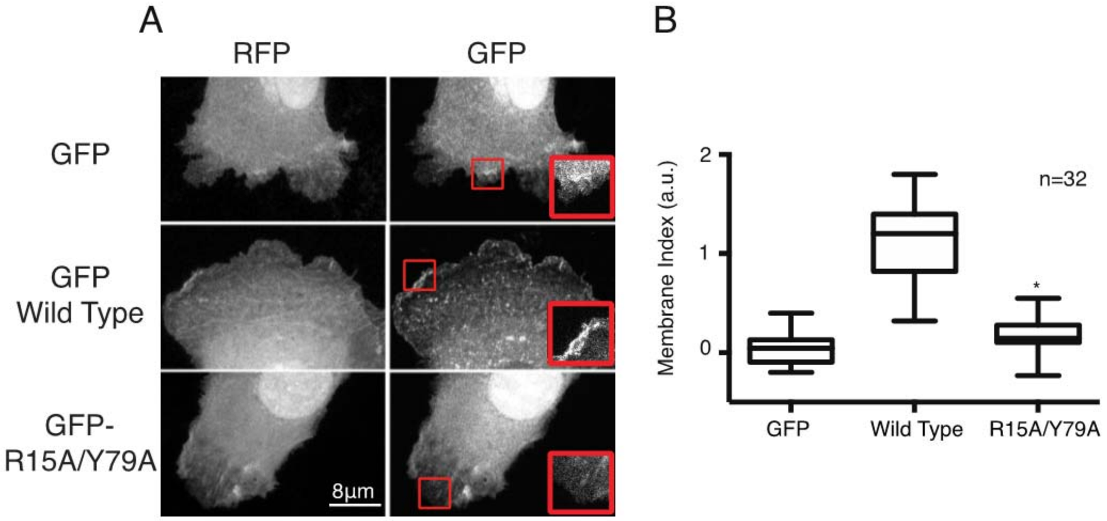

Supplementary Figure 4. CP Plasma Membrane Localization Depends on the Ability to Bind CPI Proteins. A) Confocal fluorescence images of HT-1080 cells co-expressing RFP and either GFP, GFP- CPβ2 or GFP- CPβ2R15A/Y79A Boxed regions are inset in the bottom right corner of each GFP panel. B) Quantifications of the PM index of each construct. Box-and-whisker plots indicate median, quartiles and 5th / 95th percentiles. n=32. The difference between R15A/Y79A and wild-type is statistically significant ($p < 0.01$); the difference between R15A/Y79A and GFP is not.

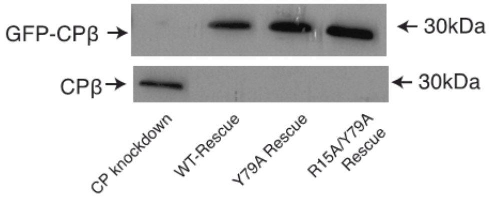

Supplementary Figure 5. Knockdown and Rescue of CPβ2. Anti- CPβ2 immunoblot documenting CP knockdown and the expression levels of wild type, single and double mutant rescue constructs
